# Beyond immersion: the cognitive mechanics of functional ludomusicology in esports performance

**DOI:** 10.3389/fpsyg.2026.1759884

**Published:** 2026-02-25

**Authors:** Xinxin Wang, Yuyi Sun

**Affiliations:** 1 The Grazyna and Kiejstut Bacewicz Academy of Music in Lodz, Łódź, Poland; 2 College of Education, Ludong University, Shandong, China

**Keywords:** arousal regulation, cognition, cognitive performance, esports, game music

## Abstract

In high-pressure esports, music is often treated as an immersion cue, yet converging evidence suggests it can also function as a cognitive ergogenic aid that modulates performance-relevant states through (i) arousal regulation, (ii) cognitive-load/attention allocation, and (iii) rhythmic entrainment. Quantitative syntheses from adjacent performance domains indicate that music produces small-to-moderate benefits in objective outcomes (e.g., a meta-analytic estimate of improved physical performance, *g* ≈ 0.31, and more positive affective valence, *g* ≈ 0.48). Evidence from sustained-attention paradigms further shows that listener-selected background music can yield measurable (albeit small) attentional gains, including faster reactions (≈7.8 ms improvement) and fewer false alarms (e.g., 0.660 vs. 0.710). Under mental-fatigue conditions, a recent systematic review reports that music can attenuate performance decrements—for example, reaction time increased in a no-music control condition (≈500 → 520 ms) but remained stable under music (≈502 → 498 ms) in a Go/NoGo task. However, benefits are boundary-conditioned: music with lyrics reliably impairs cognitive performance with small but credible effects (e.g., *d* ≈ −0.3 across memory/reading outcomes), whereas lyric-free instrumental music is often closer to null. Finally, because elite esports training and competition depend on rapid visuomotor execution, we highlight rhythmic entrainment as a plausible mechanism, while noting that tournament rules may restrict in-game music, shifting many applications to pre−/between-game windows. Together, this mini-review integrates quantitative evidence into a functional ludomusicology framework and outlines testable predictions for tailoring music by tempo, lyricality, rhythmic salience, and context (training vs. tournament).

## 1 Introduction

Esports represent a unique domain of human performance that, while physically sedentary, is a highly complex cognitive task requiring players to maintain peak mental efficiency. Success depends on the ability to perform rapid visual search, precise action execution, and real-time strategic judgment in a fast-paced, dynamic information environment. Research indicates that high-level esports involves a significant cognitive load, especially relying on sustained attention, efficient working memory management, and superior visuomotor coordination to navigate complex interfaces (Bediou et al., 2018). For instance, Multiplayer online battle arena (MOBA) and real-time strategy (RTS) games like League of Legends and StarCraft demand players to process and integrate multiple streams of visual and auditory information within milliseconds. The neural demands and physiological stress responses observed in these digital competitions are often comparable to those found in traditional high-intensity physical sports (DiFrancisco-Donoghue et al., 2019).

Despite these demands, during long training sessions, scrims, or ranked play, many players are accustomed to using non-diegetic background music (external playlists). In official tournaments, however, personal music during live gameplay may be restricted or prohibited, which constrains implementation to pre−/post-performance windows. However, the efficacy of this practice is debated; whether such music aids in cognitive regulation by inducing flow or rather acts as a source of interference that competes for neural resources remains an underexplored question. Traditional research on game music has predominantly focused on aesthetic dimensions such as “immersion” and “narrative functions.” In contrast, the emerging framework of Functional Ludomusicology proposes a utilitarian perspective, suggesting that music can also serve as a pragmatic cognitive tool. From this viewpoint, audio is not merely atmospheric but acts as a functional instrument for regulating attention allocation, physiological arousal, and fine motor rhythm control (Farkas et al., 2022; Munday 2007).

**Figure 1:**
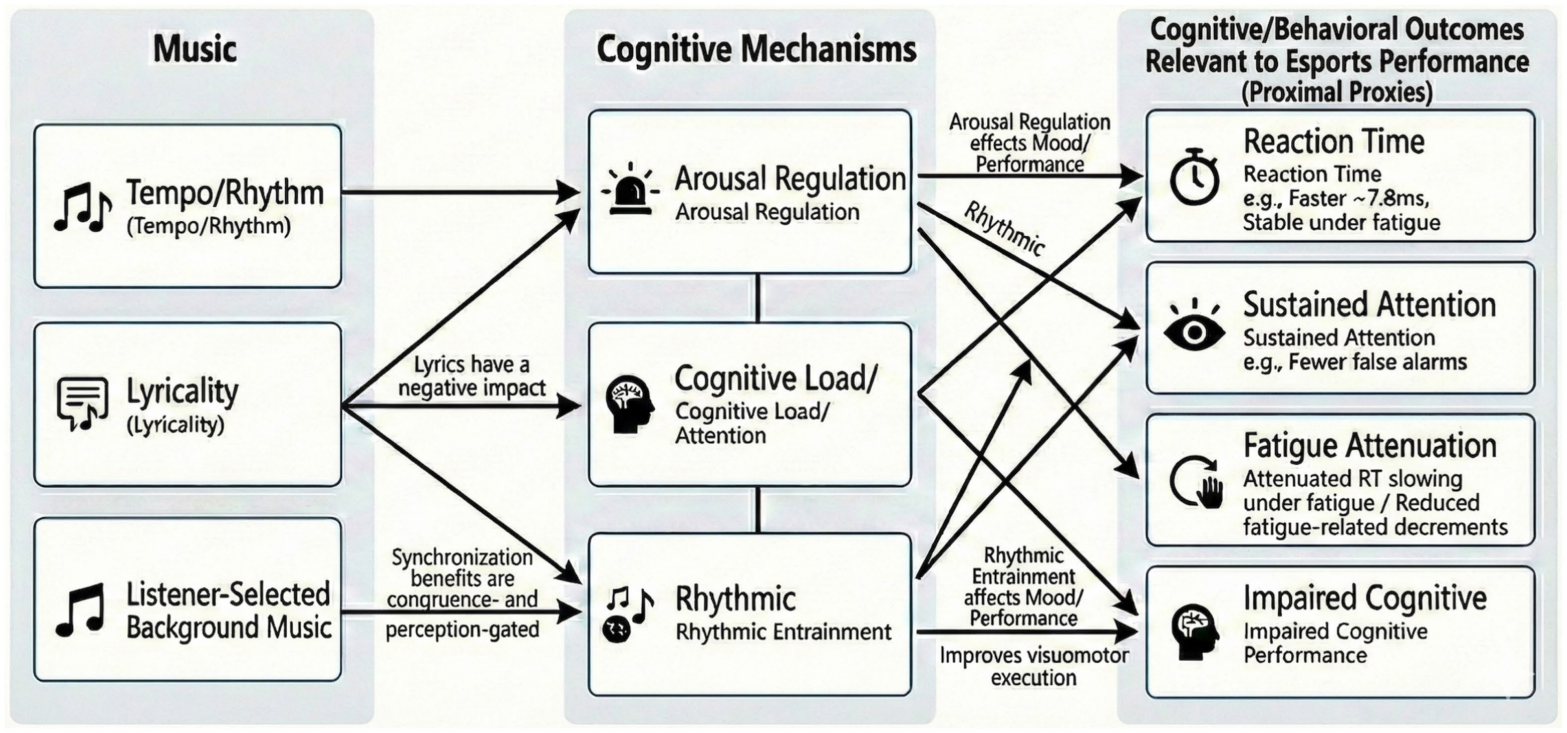
Conceptual framework linking music features to cognitive mechanisms and performance-relevant outcomes in esports.

To address this gap, this review will systematically explore the role of background music in esports through three key mechanisms. First, we examine (1) arousal regulation, explaining how music influences psychological activation levels to mitigate performance anxiety or combat fatigue based on the Yerkes-Dodson law. Second, we focus on (2) cognitive load management, analyzing the pros and cons of music in attention resource allocation, specifically how it interacts with the split-attention effect during intense gameplay. Finally, we investigate (3) rhythmic synchronization, investigating how music’s tempo can essentially “entrain” the motor system to optimize action timing and response efficiency. Through these perspectives, we aim to clarify the potential and boundaries of music as a cognitive stimulus in the esports domain. The proposed framework is summarized in Figure 1.

## 2 Arousal regulation and the Yerkes-Dodson law

Esports performance is strongly shaped by players’ psychophysiological arousal, which in turn modulates attentional control, decision speed, and fine motor stability. The Yerkes–Dodson law formalizes this relation as an inverted-U: for complex cognitive–motor tasks, performance is optimized at moderate arousal, with under-arousal linked to lapses and slowing, and over-arousal linked to anxiety, narrowed attentional focus, and motor instability (Yerkes and Dodson 1908). Meta-analytic evidence from exercise/sport contexts supports the broader claim that music can shift affective and performance-relevant states: across 139 studies (3,599 participants; 598 effect sizes), music showed small-to-moderate benefits for affective valence (*g* = 0.48, CI [0.39, 0.56]) and performance outcomes (*g* = 0.31, CI [0.25, 0.36]), with tempo emerging as a moderator (fast > slow-to-medium) for performance effects (Terry et al., 2020).

To generate testable predictions under the Yerkes–Dodson framework, this review treats tempo (beats per minute; BPM) as an engineerable proxy for a track’s arousal potential. We therefore use tempo bins as heuristics rather than hard thresholds (e.g., relaxing < 90 BPM; exciting > 120 BPM), while noting key moderators such as loudness, rhythmic salience, familiarity/preference, and baseline arousal. This operationalization aligns with recent synthesis work calling for explicit characterization of musical features (including tempo) and concluding that “exciting” versus “relaxing” music can differentially influence fatigue-related performance decrements.

### 2.1 Up-regulation: combatting fatigue and maintaining focus

Extended training blocks and repetitive ranked play can induce mental fatigue, typically expressed as reduced vigilance and impaired executive control. Here, the functional claim is not simply that “music motivates,” but that music can up-regulate arousal toward a more optimal zone and thereby mitigate fatigue-related slowing. Direct esports evidence is still limited; however, adjacent experimental literature provides converging support. A recent systematic review of music interventions under mental fatigue identified nine eligible studies (search up to Nov 18, 2023) and concluded that relaxing, exciting, and personally preferred music were each associated with reduced subjective fatigue and measurable changes in physiological markers; importantly, cognitive performance decrements in inhibition and working-memory paradigms were countered by both relaxing and exciting music, and exciting music appeared more effective for reducing reaction time in working-memory tasks (Ding et al., 2025).

This pattern is consistent with the broader exercise/sport meta-analysis showing a small ergogenic effect of music on performance and identifying tempo as a moderator (fast > slow-to-medium) (Terry et al., 2020). What this adds to the framework is a phase-specific prediction: during under-arousal states (late-session fatigue), higher-tempo, rhythmically salient, non-lyrical music should be most likely to support faster responding and sustained engagement, whereas the same stimulation may be counterproductive in already high-arousal moments. Methodologically, a key limitation is ecological validity: most fatigue paradigms use controlled tasks rather than competitive esports workloads, so esports-specific tests should treat this evidence as mechanistic justification rather than definitive effect estimation (Ding et al., 2025; Terry et al., 2020).

### 2.2 Down-regulation: mitigating anxiety in high-stakes moments

Competitive play can also drive over-arousal, increasing the risk of attentional narrowing and “choking.” Because tournament rules may restrict in-game music, down-regulation protocols are most realistic in pre-match and between-map/round recovery windows. Physiological evidence supports tempo-linked autonomic shifts: in a controlled laboratory study (12 practicing musicians and 12 age-matched controls), Bernardi et al. (2006) measured cardiovascular/respiratory variables during exposure to multiple music styles plus a randomly inserted pause, and found that faster tempi increased ventilation, blood pressure, and heart rate relative to baseline, whereas the pause reduced heart rate, blood pressure, and ventilation even below baseline; critically, effects depended more on tempo than on musical style. What this adds to the framework is a concrete recovery mechanism: slower, more predictable tracks (or strategically designed “silence/pause” periods) are plausible tools for parasympathetic rebound during breaks, while avoiding the false assumption that any music is calming. This also clarifies an apparent cross-study inconsistency: the sport/exercise meta-analysis found no reliable benefit of music on heart rate overall (*g* = 0.07, CI [−0.03, 0.16]), suggesting that heart-rate effects are context- and protocol-dependent (resting listening vs. exertion; fast vs. slow; inclusion of pauses), and should not be treated as a uniform mediator (Ding et al., 2025; Bernardi et al., 2006).

### 2.3 Individual differences and personalized strategies

Arousal regulation is not one-size-fits-all. Individual baseline arousal and personality can moderate whether music pushes a player toward—or away from—the Yerkes–Dodson optimum. For example, Cassidy & MacDonald used a between-participants design with 40 undergraduates assigned to one of four background conditions (high-arousal/negative-affect music; low-arousal/positive-affect music; everyday noise; silence). They reported that performance was moderated by internal arousal: introverts appeared more detrimentally affected by high-arousal music and noise, relative to extraverts (Cassidy and MacDonald 2007). What this adds to the framework is a moderator specification rather than a generic claim: (i) baseline arousal/personality should interact with tempo/arousal potential, and (ii) individualized prescriptions are theoretically warranted—especially when interventions are used for up-regulation (fatigue) versus down-regulation (stress). Methodologically, this also flags a measurement need for esports studies: to model person × music × phase interactions, researchers should measure baseline arousal (e.g., HRV), trait differences, and situational demands rather than only relying on self-reported preference (Ding et al., 2025; Cassidy and MacDonald 2007).

## 3 Cognitive load and attention resource allocation

In high-intensity esports environments, players operate under extreme multitasking demands: they must simultaneously process complex visual cues, maintain real-time strategic communication with teammates, and execute rapid mechanical actions. In this saturated cognitive landscape, background music can be a double-edged sword—it may facilitate performance by stabilizing mood/arousal (often discussed under the umbrella of the “Mozart effect” literature; see Quon et al., 2021), but it can also introduce interference by consuming scarce attentional and working-memory resources, consistent with the “irrelevant speech effect” and related attentional-resource accounts (Elliott and Briganti 2012). In the proposed cognitive framework, the key question is therefore not whether music is “good” or “bad,” but when it reduces distraction (e.g., masking irregular noise; reducing mind-wandering) versus when it increases extraneous load (e.g., semantic competition from lyrics; masking task-relevant auditory signals).

### 3.1 The finite nature of attention and channel conflict

According to limited-capacity accounts of attention, cognitive resources are finite and behave as a constrained pool; when primary-task demands are high, additional stimuli compete for processing capacity (Kahneman 2003). This competition becomes most costly when background audio shares processing channels with task-relevant operations. Critically, when background music contains intelligible lyrics, it can trigger automatic linguistic/semantic processing and thereby create channel conflict with verbal working memory and communication. Empirical evidence supports this mechanism: in a within-subject design (Session 1 *n* = 123; Session 2 *n* = 113), participants performed multiple cognitive tasks under silence, instrumental lo-fi, and music with lyrics, and lyrical music produced reliable performance costs relative to silence (e.g., verbal recall *d* = −0.32; visual recall *d* = −0.33; reading comprehension accuracy *d* = −0.19), whereas instrumental music showed smaller and less consistent decrements (e.g., verbal recall *d* = −0.16; visual recall *d* = −0.23) (Souza and Leal Barbosa 2023).

Framework contribution: this body of evidence adds a *mechanism-specific* constraint: the main risk is not generic “sound distraction,” but semantic competition. Accordingly, for communication-heavy esports (e.g., Multiplayer Online Battle Arena [MOBA] games), the framework predicts that lyrical music will be most disruptive during phases with high comm density and rapid verbal updating (Souza and Leal Barbosa 2023; Elliott and Briganti 2012).

### 3.2 The masking effect and environmental control

Not all auditory input is detrimental. In noisy training environments (keyboard clatter, crowd noise, ambient chatter), unpredictable sounds can trigger orienting responses and attentional capture. In such contexts, predictable, non-lyrical music can act as a “sonic wall,” masking irregular distractions and reducing subjective disturbance (Schlittmeier and Hellbrück 2009). Complementing this account, recent sustained-attention evidence suggests that preferred background music can yield small but measurable improvements in vigilance: in two experiments using a Psychomotor Vigilance Task, background music was associated with faster reaction times (Experiment 1: *N* = 106, *b* = 7.785 ms, *p* = 0.047; Experiment 2: *N* = 77, *b* = 8.100 ms, *p* = 0.030) and fewer false alarms in a noise baseline (music-present *M* = 0.660 vs. music-absent *M* = 0.710, *p* = 0.004) (Kiss and Linnell 2024).

Cross-study comparison. These findings help reconcile an apparent inconsistency with Section 3.1: tasks that strongly rely on encoding and verbal processing show clear lyrical costs (Souza and Leal Barbosa 2023), whereas vigilance tasks can show small gains when music is preferred and the core challenge is sustaining alertness (Kiss and Linnell 2024).

Framework contribution. This evidence adds a *benefit pathway* to the framework: under some conditions, music may reduce off-task thought and stabilize attentional state (Kiss and Linnell 2024), particularly in noisy settings where masking reduces irregular distraction (Schlittmeier and Hellbrück 2009). However, it also implies boundary conditions related to volume, controllability, and task phase.

### 3.3 Genre-specific tolerances and strategy

The impact of music is also moderated by game genre and the degree of auditory-signal dependence. First-person shooter (FPS) games are typically “low-tolerance” contexts because survival and competitive advantage can depend on precise auditory localization (e.g., footsteps, reload cues). In such settings, music plausibly masks task-relevant audio signals, increasing extraneous load. By contrast, in visually dominant genres where immediate success depends less on auditory micro-cues, moderate background music may have lower functional cost and can influence decision style and overall experience. For example, in an experimental game study (*N* = 60), background music affected risk-taking and player-experience metrics, with effects especially apparent early in play (Rogers et al., 2019).

Framework contribution. This evidence adds a genre-level boundary condition: any attentional-stabilization benefits of music (Section 3.2) can be outweighed when the primary task requires fine-grained auditory perception, implying that music recommendations must be conditional on auditory cue reliance (Rogers et al., 2019).

Section summary. Taken together, the cognitive-load literature supports a conditional model: (i) lyrics increase extraneous load via semantic competition, especially under high communication demands (Souza and Leal Barbosa 2023; Elliott and Briganti 2012); (ii) preferred, predictable music can modestly stabilize vigilance and reduce distraction in some contexts (Kiss and Linnell 2024; Schlittmeier and Hellbrück 2009); and (iii) benefits are most likely to collapse in genres where auditory signals are performance-critical (Rogers et al., 2019). This synthesis converts “music helps/hurts” into testable predictions aligned with the proposed framework.

## 4 Rhythmic entrainment and motor timing

Rhythmic entrainment provides a mechanistic route by which music can shape esports micro-timing: an external beat can serve as a temporal scaffold that stabilizes internal prediction of “when” to act, thereby supporting fine-grained visuomotor execution (e.g., kiting/orb-walking in MOBAs, recoil patterns in FPS, frame-tight inputs in fighting games). Importantly, the strongest evidence for this pathway comes from converging behavioral and neuroimaging work showing that beat-based rhythms recruit motor-related cortico-striatal circuitry even without overt movement, consistent with the idea that rhythm perception partially “pre-activates” the motor system (Grahn and Brett 2007; Grahn and Rowe 2009).

In a controlled reproduction paradigm, Grahn and Brett (2007) tested adults’ ability to reproduce rhythmic sequences after listening. With *N* = 20, rhythms designed to induce a regular beat (“metric simple”) were reproduced more accurately than both “metric complex” and “nonmetric” rhythms (metric simple vs. metric complex: *t* (19) = 5.47, *p* < 0.001; metric simple vs. nonmetric: *t* (19) = 5.24, *p* < 0.001), with a large within-participant effect size (approx. *d*_x_ ≈ 1.22 and 1.17, respectively) (Grahn and Brett 2007). In contrast, metric complex vs. nonmetric was not significant (*p* = 0.19), suggesting that not all structured rhythms yield the same timing benefit (Grahn and Brett 2007).

Crucially, this behavioral advantage was mirrored by neuroimaging evidence: in a separate fMRI study (*N* = 27; 14 musicians/13 nonmusicians), beat-inducing rhythms elicited higher activity in the basal ganglia and SMA, consistent with a motor-network contribution to beat perception and internal timing prediction (Grahn and Brett 2007).

These results justify the paper’s claim that rhythm can operate as a functional timing scaffold—it improves temporal reproduction behaviorally and is implemented neurally in cortico-striatal/SMA circuitry that is directly relevant to timing and action sequencing (Grahn and Brett 2007).

Extending this logic, Grahn and Rowe (2009) used fMRI to separate conditions requiring stronger external beat marking (volume accents), weaker accents (duration), versus internally generated beat (unaccented). Across *N* = 36 (19 musicians, 17 nonmusicians), behavioral beat ratings showed a very strong main effect of beat (*F* (1, 34) = 115.51, *p* < 0.001; *η*p^2^ ≈ 0.77), indicating that participants reliably distinguished beat vs. nonbeat sequences (Grahn and Rowe 2009).

At the neural level, beat (vs. no-beat) most robustly activated the putamen bilaterally (e.g., peak *t* scores in Table 1 include *t* = 5.20 in left posterior putamen and *t* = 5.01 in right anterior putamen), and beat conditions increased functional coupling between anterior putamen and motor/auditory regions (e.g., PPI coefficients showing higher coupling in beat vs. nonbeat for right SMA: 0.048 → 0.060; left SMA: 0.040 → 0.056; plus PMC/STG involvement at *p* ≤ 0.05 SVC in Experiment 1) (Grahn and Rowe 2009).

**Table 1:** Summary of key studies linking background music features to cognitive mechanisms and performance-relevant outcomes.

Evidence source	Design/sample	Music manipulation	Task/context	Main outcomes	Direction & magnitude	What it adds
Terry et al. (2020)	Meta-analysis (*k* = 139; *N* = 3,599; 598 ES)	Music across sport/exercise tasks	Physical performance paradigms	Performance; affect	Performance *g* = 0.31; valence *g* = 0.48; tempo moderates (fast>slow)	Supports arousal-regulation plausibility; tempo as moderator
Ding et al. (2025)	Systematic review (*k* = 9 studies)	Relaxing/exciting/preferred music	Mental-fatigue paradigms	Fatigue; RT/WM; physiology	Review-level conclusion: music mitigates fatigue effects; exciting often stronger for RT/WM	Justifies fatigue-window up-regulation logic; motivates BPM bins
Souza and Leal Barbosa (2023)	Within-subject (*n* = 123; *n* = 113)	Silence vs. instrumental vs. lyrics	Memory/reading/arithmetic	Accuracy across tasks	Lyrics impair: verbal recall *d* = −0.32; visual recall *d* = −0.33; reading *d* = −0.19 (instrumental smaller)	Pins “semantic competition” mechanism; predicts lyrics × comm-load interaction
Kiss and Linnell (2024)	Experiments (*N* = 106; *N* = 77)	Preferred music vs. no music	PVT sustained attention	RT; false alarms; thought probes	RT faster: *b* ≈ 7.8 ms (*p* = 0.047) & *b* ≈ 8.1 ms (*p* = 0.030); false alarms 0.660 vs. 0.710 (*p* = 0.004)	Supports attentional anchoring/masking benefit under vigilance/noise
Grahn and Brett (2007)	Lab + fMRI (*N* = 20 behavior; *N* = 27 fMRI)	Metric simple vs. complex vs. nonmetric	Rhythm reproduction; beat perception	Timing accuracy; motor network activity	Metric simple reproduced better: *t* (19) = 5.47 & 5.24; motor circuitry (SMA/basal ganglia)	Establishes beat as temporal scaffold; motor-network recruitment
Grahn and Rowe (2009)	fMRI (*N* = 36)	Beat marking vs. internally generated beat	Beat perception	Beat ratings; putamen activation/coupling	Beat effect huge: *F*(1,34) = 115.51; *η*p^2^ ≈ 0.77; coupling differences	Adds “internal prediction” nuance; supports congruence boundary
Albert et al. (2022)	Interactive VR study (*N* = 54)	Sync vs. nonsync	Beat Saber	Performance; workload; experience	Sync improves performance/fluency and reduces workload; effects perception-gated	Ecological link: entrainment → execution & workload, moderated by perception
Schlittmeier and Hellbrück (2009)	Lab study	Background music as noise abatement	Office-like noise	Performance & preference	(Use qualitative if you do not extract numbers)	Supports masking mechanism under unpredictable noise
Rogers et al. (2019)	Game experiment (*N* = 60)	Background music manipulation	Risk-taking & experience	Risk metrics; experience	Effects early in play (qual/quant per paper)	Adds genre/task dependence for decision-policy outcomes

However, an instructive inconsistency emerged: subjective beat ratings followed roughly volume beat > unaccented beat > duration beat, whereas putamen activity followed unaccented > duration > volume, i.e., striatal responses did not simply track perceived beat salience (Grahn and Rowe 2009). This pattern supports an interpretation that the putamen may be especially engaged when timing must be internally generated/predicted, not merely when the beat is externally obvious (Grahn and Rowe 2009). The authors also note that some connectivity effects were less robust in the second experiment due to variability/limited power, underscoring that entrainment-relevant coupling is detectable but method-sensitive (Grahn and Rowe 2009).

This study adds a key boundary condition: rhythm benefits should be strongest when the auditory beat helps the player’s system generate stable internal timing predictions—and the mechanism is not purely “more salient beat = better,” but rather “beat that supports prediction/generation = better,” which directly motivates your congruence vs. incongruence argument (Grahn and Rowe 2009).

Translating beat-based scaffolding into interactive play contexts, Albert et al. (2022) tested synchronization effects in the VR rhythm game *Beat Saber* with *N* = 54 and found that synchronized versus non-synchronized music–gameplay conditions produced significant differences in performance and perceived workload, alongside changes in player experience; importantly, effects were strongly moderated by whether participants could consciously perceive the synchronization differences (Albert et al., 2022).

This provides preliminary task-ecological support that synchronization can affect objective execution and subjective load, but it also highlights a practical constraint highly relevant for esports: entrainment benefits may be perception-gated (rhythmic salience/clarity and the player’s sensitivity), which should be treated as a moderator in your model (Albert et al., 2022).

Taken together, these literatures converge on a coherent functional claim: (i) beat-based rhythms improve temporal reproduction and recruit SMA/basal ganglia networks central to timing prediction (Grahn and Brett 2007), (ii) striatal engagement and cortico-striatal connectivity increase when internal beat generation/prediction is required, suggesting a mechanism for how rhythm can stabilize micro-timing under uncertainty (Grahn and Rowe 2009), and (iii) in interactive tasks, synchronization can reduce workload and improve performance, but only when synchronization is perceptible (Albert et al., 2022).

Accordingly, in esports we predict that rhythmically salient, tempo-stable, low-lyric tracks will most plausibly support repetitive periodic mechanics (e.g., orb-walking cycles, recoil cadence training) by stabilizing internal timing, whereas incongruent or weakly perceived rhythms may increase temporal conflict and attentional switching—reducing precision and fragmenting action sequences (Albert et al., 2022; Grahn and Brett 2007; Grahn and Rowe 2009).

## 5 Discussion

Key empirical findings and their mapping to the proposed mechanisms are summarized in Table 1. This paper outlines three key mechanisms by which background music influences esports performance: arousal regulation, attention allocation, and operational rhythm optimization. While existing research provides theoretical support for music as a cognitive regulation tool, its systematic application in esports is still in its early stages.

### 5.1 Practical implications: the boundary between training and tournament

When applying these mechanisms to practice, it is crucial to recognize the distinction between training environments and competitive play. Official tournaments may restrict or prohibit personal background music during live gameplay, limiting direct in-game implementation. Accordingly, lyric-free and tempo-targeted recommendations are most applicable to training, scrims, and non-official ranked play, where players can control their auditory environment. In tournament settings, music interventions should be framed primarily as state preparation and recovery tools outside active gameplay (e.g., pre-match warm-up, between maps/rounds, breaks, or technical pauses), contingent on event rules. This distinction improves ecological validity and prevents conflating professional competition constraints with everyday play contexts.

### 5.2 Future directions

Based on the theoretical framework and practical constraints discussed above, future research and practice can be expanded in the following directions.

#### 5.2.1 Personalized audio prescriptions

Individuals exhibit significant differences in their response to music, influenced by personality, experience, and preferences. For example, introverted players may be more sensitive to high-BPM music and easily distracted by it, while extroverts might find it motivating. Future research could combine personality assessments and behavioral responses to create personalized music playlists for different pre-competition situations (calm start, motivational activation, rhythm balancing). This “psychological soundtrack” would transform music into a targeted psychological intervention tool.

#### 5.2.2 Adaptive music systems

With the development of AI and wearable devices, real-time monitoring of players’ physiological and cognitive states has become possible. Future designs could include music systems based on EEG (electroencephalography) or HRV (heart rate variability) that dynamically adjust music tempo, volume, and style to maintain the player in an optimal “flow window.” For example, when the system detects a drop in attention or an emotional spike, it could automatically switch to a smoother or more motivating music track, providing real-time cognitive intervention.

#### 5.2.3 Methodological innovations and enhancing ecological validity most

Current studies on music and cognitive performance rely on simplified models such as button-press tasks or short-duration tests, which fail to replicate the real esports environment. Future studies should adopt tools with higher ecological validity, such as fNIRS (functional near-infrared spectroscopy) or eye-tracking, to capture the effects of music interventions on brain activation, attention shifts, and decision-making in real gaming environments. Long-term intervention effects should also be examined, rather than just short-term exposure effects.

## 6 Conclusion

The role of music in esports has expanded beyond emotional enhancement or background decoration; it now encompasses multidimensional functions that regulate arousal, optimize attention allocation, and enhance operational rhythm. As a “Cognitive Ergogenic Aid”, functional music can, through scientific design, effectively improve players’ competitive state and psychological resilience. This paper calls for coaches, sports psychologists, and team managers to integrate sound environment design into regular training programs, combining personalized and task-matching strategies to fully harness music’s cognitive potential. With ongoing technological advancements and interdisciplinary integration, music intervention is set to play an increasingly pivotal role in optimizing esports performance.
